# Morphological and genomic characteristics of two novel actinomycetes, *Ornithinimicrobium sufpigmenti* sp. nov. and *Ornithinimicrobium faecis* sp. nov. isolated from bat faeces (*Rousettus leschenaultia* and *Taphozous perforates*)

**DOI:** 10.3389/fcimb.2023.1093407

**Published:** 2023-02-14

**Authors:** Yuyuan Huang, Suping Zhang, Yuanmeihui Tao, Jing Yang, Shan Lu, Dong Jin, Ji Pu, Wenbo Luo, Han Zheng, Liyun Liu, Jia-fu Jiang, Jianguo Xu

**Affiliations:** ^1^ State Key Laboratory of Infectious Disease Prevention and Control, National Institute for Communicable Disease Control and Prevention, Chinese Center for Disease Control and Prevention, Beijing, China; ^2^ Department of Epidemiology, Center for Global Health, School of Public Health, Nanjing Medical University, Nanjing, Jiangsu, China; ^3^ Research Units of Discovery of Unknown Bacteria and Function, Chinese Academy of Medical Sciences, Beijing, China; ^4^ Beijing Institute of Microbiology and Epidemiology, State Key Laboratory of Pathogen and Biosecurity, Beijing, China; ^5^ Research Institute of Public Health, Nankai University, Tianjin, China

**Keywords:** *Ornithinimicrobium sufpimenti*, *Ornithinimicrobium faecis*, bats, genome characteristics, carotenoids, antibiotic sensitivity

## Abstract

Four Gram-staining-positive, aerobic, non-motile, circle-shaped bacteria were isolated from the faeces of bats (*Rousettus leschenaultia* and *Taphozous perforates*) collected from Guangxi autonomous region (E106°49′20″, N22°20′54″) and Yunnan province (E102°04′39″, N25°09′10″) of South China. Strains HY006^T^ and HY008 shared highly 16S rRNA gene sequence similarity to those of *Ornithinimicrobium pratense* W204^T^ (99.3%) and *O. flavum* CPCC 203535^T^ (97.3%), while the strains HY1745 and HY1793^T^ were closest to the type strains *O. ciconiae* H23M54^T^ (98.7%), *O. cavernae* CFH 30183^T^ (98.3%), and *O. murale* 01-Gi-040^T^ (98.1%). Furthermore, when compared to the other members of the genus *Ornithinimicrobium*, the digital DNA-DNA hybridization and average nucleotide identity values of the four novel strains were within the ranges of 19.6–33.7% and 70.6–87.4%, respectively, both of which were below the respective recommended cutoff values of 70.0% and 95–96%. Significantly, strain HY006^T^ was resistant to chloramphenicol and linezolid whereas strain HY1793^T^ was resistant to erythromycin, clindamycin (intermediately), and levofloxacin (intermediately). The main cellular fatty acids (>20.0%) of our isolates were *iso*-C_15:0_ and *iso*-C_16:0_. Strains HY006^T^ and HY1793^T^ contained ornithine as the diagnostic diamino acid, also along with the alanine, glycine and glutamic acid in their cell wall. Based on phylogenetic, chemotaxonomic and phenotypic analyses, these four strains could be classified as two novel species of the genus *Ornithinimicrobium*, for which the names *Ornithinimicrobium sufpigmenti* sp. nov. and *Ornithinimicrobium faecis* sp. nov. are proposed. The type strains are HY006^T^ (=CGMCC 1.16565^T^ =JCM 33397^T^) and HY1793^T^ (=CGMCC 1.19143^T^ =JCM 34881^T^), respectively.

## Introduction

Bats (order Chiroptera), only a group of flight-capable mammals, have sparked global attention due to their possible zoonotic association with severe acute respiratory syndrome coronavirus (SARS-CoV) and severe acute respiratory syndrome coronavirus 2 (SARS-CoV-2) (Ge et al., 2013; Zhou et al., 2020; Hu et al., 2021). Furthermore, many new viruses, such as adenovirus, hantaviruses, and Shimoni bat virus, have been discovered in bats (Kuzmin et al., 2010; Witkowski et al., 2014; Iglesias-Caballero et al., 2018). However, previous studies have primarily focused on viruses borne by bats (such as *Artibeus lituratus*, *Pteropus giganteus*, *P. vampyrus*, and so on.), leaving the prevalence and abundance of potentially pathogenic bacteria in bats largely neglected (Tan et al., 2021). Significantly, diverse filoviruses were detected in the visceral organs of *Rousettus* sp. bats, which were collected in Yunnan province (P.R. China) in 2009 and 2015 (Yang et al., 2017). In addition, *Toxoplasma gondii* infection was easily found in the *Taphozous melanopogon* collected from Guangxi, China (Jiang et al., 2014). We are curious if bat fecal samples contain any novel bacteria alongside the viruses and parasites. Interestingly, some novel bacterial species previously isolated from feces of the *Rousettus leschenaultia* and/or *Taphozous perforates* in our laboratory belong to the phylum Actinobacteria, such as *Brevibacterium zhoupengii* (Huang et al., 2022a), *Microbacterium fandaimingii* (Zhou et al., 2021), *Tomitella gaofuii* and *T. fengzijianii* (Huang et al., 2022d). In the present study, we continue to explore bat fecal microbiota and describe the phenotypic and genomic characterization of a novel species in the genus *Ornithinimicrobium* belonging to phylum Actinobacteria.

The genus *Ornithinimicrobium* which currently accommodates 14 validly published species (https://lpsn.dsmz.de/search?word=Ornithinimicrobium) was described by Groth et al. (Groth et al., 2001) and belongs to the family *Ornithinimicrobiaceae* (Nouioui et al., 2018) within the order *Micrococcales*. Strains belonging to the genus *Ornithinimicrobium* have been isolated from various sources, including soil (Groth et al., 2001; Liu et al., 2008), plants (Ramaprasad et al., 2015; Fang et al., 2017), water (Liu et al., 2013), and faeces of birds (Lee et al., 2021). *Ornithinimicrobium* is the genus of aerobic or facultatively anaerobic, non-motile Gram-stain-positive, catalase-positive, and oxidase-negative, with 68.3–72.5 mol% DNA G+C contents. The cellular fatty acid profile is dominated by *iso*-branched-chain acids (Fang et al., 2017; Zhang et al., 2019) and MK-8 (H_4_) is the major menaquinone. It is worth noting that *O. kibberense* was first isolated from the Indian Himalayas by Mayilraj in 2006 (Mayilraj et al., 2006), which showed high levels of cytotoxic activity against the murine mammary cell carcinoma 4T1 and human mammary adenocarcinoma MCF-7 cell lines in 2017 (Diaz-Cardenas et al., 2017). Additionally, *O. pekingense* was first isolated from the activated sludge by Liu in 2008 (Liu et al., 2008), which may be regarded as a causative agent for causing ocular infection (Borsali et al., 2011). These studies were in line with our previously proposed reverse microbial etiology (Xu, 2019), which was to predict and prevent a potential emerging infectious disease caused by an unknown microorganism. Moreover, *Ornithinimicrobium* may be used as one of the potential markers to distinguish Graves’ disease and Hashimoto’s thyroiditis from the healthy population for participating in the occurrence and development of the disease by regulating purine metabolism and pyrimidine metabolism (Zhao et al., 2022).

Here we report the polyphasic characterization of two novel species (designed strains HY006^T^/HY008 and HY1745/HY1793^T^) were isolated from the faeces of bats (*Rousettus leschenaultia* and *Taphozous perforates*), for which the names *Ornithinimicrobium sufpigmenti* sp. nov. and *Ornithinimicrobium faecis* sp. nov. are proposed, respectively. Strains HY006^T^ (=CGMCC 1.16565^T^ =JCM 33397^T^) and HY1793^T^ (=CGMCC1.19143^T^ =JCM 34881^T^) were obtained the certificates of deposition and availability from the China General Microbiological Culture Collection Center (CGMCC) and the Japan Collection of Microorganisms (JCM), respectively. Furthermore, MK-8(H_4_) was detected as the predominant menaquinone in strains HY006^T^ and HY1793^T^, and the whole-cell peptidoglycan of both strains contained ornithine, alanine, glycine, and glutamic acid. They also contained glucose in the whole cell sugar. Significantly, strain HY006^T^ was resistant to chloramphenicol, linezolid, and intermediately resistant to erythromycin and tetracycline, while strain HY1793^T^ was resistant to erythromycin, intermediately resistant to clindamycin and levofloxacin. In addition, *O. sufpigmenti* HY006^T^ accumulated two different carotenoids at significant concentrations by extraction with a solvent system while *O. faecis* could not detect them.

## Materials and methods

### Sample collection and strain isolation

The fecal samples from two bat genera were obtained from the Wuhan Institute of Virology, Chinese Academy of Sciences, which were collected from different far-apart sites in Yunnan and Guangxi provinces in October 2013 and July 2011, respectively. The specific latitude and longitude are E102°04′39″, N25°09′10″ (Yunnan) and E106°49′20″, N22°20′54″ (Guangxi) (**Table S1**). In addition, the species of bats have been identified as *Rousettus leschenaultia* and *Taphozous perforates* by blasting the cytochrome b gene sequence (Huang et al., 2022c). Sample collection, transportation and storage were almost like what was delineate antecedently (Ge et al., 2012; Huang et al., 2022a). Briefly, the clean plastic sheets (around 4 square meters) were placed under known bat roosting sites in their natural habitat (usually caves) at approximately six o’clock in the afternoon, and the fecal samples were collected from the sheets in the next morning (approximately 6:00 am) and placed into sterile tubes.

The samples were transported to the laboratory and stored at –80°C until use. Fecal samples obtained from the same bat species were mixed in the laboratory to minimize variations within the same species. The isolation of bacteria from the animal feces was conducted as described previously (Bai et al., 2016; Wang et al., 2018). Briefly, approximately 1 gram of the fecal sample was added in 1 mL of 0.85% (w/v) NaCl solution by lightly whirlpool oscillation, and 150 µL of the suspension was spread on brain-heart infusion (BHI) agar. After incubation at 28°C for 7 days under aerobic, emerging colonies of different morphology were selected, purified, and preliminarily identified by PCR amplification and sequencing of 16S rRNA gene sequence analysis. As previously described (Wang et al., 2018), the nearly full-length 16S rRNA gene fragments (1510 bp) of the four strains were obtained from TA clones using the *pEASY*-T3 cloning kit (Transgenes) after amplification with two bacterial universal primers, 27F and 1492R (Jin et al., 2013). The 16S rRNA gene sequences of our isolates were deposited in GenBank and calculated 16S rRNA gene sequence similarities with public 16S rRNA gene sequences of type strains using EzBioCloud (https://www.ezbiocloud.net/) 16S based ID service (Yoon et al., 2017a). Among these isolates, four circular, convex, smooth colonies (designated HY006^T^, HY008, HY1745, and HY1793^T^), undergoing an identical procedure as other colonies, were identified as potential novel species of genus *Ornithinimicrobium* and preserved at –80°C in BHI broth supplemented with glycerol (20%, v/v) for further analysis. Strains HY006^T^ and HY1793^T^ were isolated from feces of *Rousettus leschenaultia* whereas HY008 and HY1745 were isolated from feces of *Taphozous perforates*. According to the Rules 15, 18a, and 18b from Chapter 3 of International Code of Nomenclature of Prokaryotes (Charles et al., 2019), strains HY006^T^ and HY1793^T^ were designated as the type strain, both of them were deposited in the China General Microbiological Culture Collection Center (CGMCC) and the Japan Collection of Microorganisms (JCM) from which they would be available.

### Phylogenetic analyses based on the 16S rRNA gene sequences

The assembled sequences were aligned with representative 16S rRNA gene sequences of phylogenetically related species using ClustalW software (Thompson et al., 1994). Phylogenetic trees were reconstructed by the type strain of the family *Ornithinimicrobiaceae* with three algorithms, neighbor-joining (Saitou and Nei, 1987), maximum-likelihood (Guindon and Gascuel, 2003) and maximum-parsimony (Kolaczkowski and Thornton, 2004), using MEGA version X (Kumar et al., 2018) based on bootstrap analysis with 1000 replications, as previously described (Huang et al., 2019; Zhu et al., 2019). *Arthrobacter globiformis* JCM 078085^T^ (=NBRC 12137^T^) was used as the outgroup.

### Whole-genome sequencing analyses

The genomic DNA of four strains was extracted using the Wizard Genomic DNA Purification Kit (Promega) according to the manufacturer’s instruction. Strains HY006^T^ and HY1793^T^ were sequenced by single molecule real-time (SMRT) technology (McCarthy, 2010) on the Pacific Biosciences (PacBio) sequencing platform, which was assembled using *de novo* and analyzed using the Hierarchical Genome Assembly Process (HGAP4) application (Castanera et al., 2019) to obtain its whole genome sequence without gaps. Meanwhile, the draft genome sequences of strains HY008 and HY1745 were sequenced on the Illumina HiSeq TM2000 platform and assembled using VELVET (Zerbino and Birney, 2008). The number of tRNAs, rRNAs, and protein-coding DNA sequences (CDSs) were predicted using GeneMarkS+4.2 (Berlin et al., 2015). Gene calling and annotation were performed with the Rapid Annotation using Subsystem Technology (RAST) server (https://rast.nmpdr.org/) (Aziz et al., 2008) and the SEED viewer framework (Overbeek et al., 2014). In addition, the antibiotic resistance genes of strains are obtained by comparison in the comprehensive antibiotic resistance database (https://card.mcmaster.ca/analyze) (Alcock et al., 2022). Using the virulence factor database (VFDB, http://www.mgc.ac.cn/VFs/main.htm) (Liu et al., 2019), we identified genome encoded virulence factor (VF) genes. The database of Clusters of Orthologous Groups of proteins (COGs) is a phylogenetic classification of proteins encoded in completely sequenced genomes and the COGs comprise a framework for functional and evolutionary genome analysis (Tatusov et al., 1997). Furthermore, The average nucleotide identity (ANI Calculator | Ezbiocloud.net; with a threshold of 95–96%) and digital DNA-DNA hybridization (dDDH) (http://ggdc.dsmz.de/ggdc.php; with a threshold of 70%) values of the whole genomes of our four isolates with closely related type strains were calculated using the OrthoANIu algorithm (Yoon et al., 2017b) and Genome-to-Genome Distance Calculator 3.0 (GGDC 3.0) (Meier-Kolthoff et al., 2022), respectively.

To further validate the taxonomic status of the four strains in the genus *Ornithinimicrobium*, a phylogenomic tree based on core genes was constructed using the FastTree program (Price et al., 2009). The core genes were extracted from 22 whole genomes (**Table S2**) from the NCBI GenBank database (including genomes of strains HY006^T^, HY008, HY1745, HY1793^T^, *Arthrobacter globiformis* NBRC 12137^T^ serving as outgroup and 17 available genomes within the family of *Ornithinimicrobiaceae*) as aligned by Mafft and determined by CD-HIT (Fu et al., 2012) software (protein sequence identity threshold, 0.4). The output result was visualized in Dendroscope 3 (version 3.5.10) (Huson and Scornavacca, 2012) and modified by Interactive Tree of Life (https://itol.embl.de/) (Letunic and Bork, 2021). Moreover, the whole-genome comparison and annotation of orthologous clusters across multiple species were conducted with OrthoVenn2 (Xu et al., 2019). The pairwise and multiple whole genome alignments were generated with the progressiveMauve algorithm (version 2015-02-26) (Darling et al., 2010).

### Phenotypic and biochemical characterization

The phenotypic and physiological tests were similar to our previously described (Huang et al., 2022a). Bacterial growth test was determined at a range of temperatures (4, 10, 15, 20, 25, 28, 30, 35, 37, 40, and 45°C) in BHI broth for 7 days. The pH levels (4.0–11.0, at intervals of 0.5) and NaCl concentrations (0–12%, w/v, at intervals of 0.5%) for growth were also examined using BHI broth at 28°C. The cells have an incubation period of up to 7 days for observing growth under microaerophilic (5.0% CO_2_ incubator) or anaerobic conditions (80.0% N_2_, 10.0% CO_2_, and 10.0% H_2_) by incubating the strains on identical plates. Cell morphology was observed by microscope (Light microscope, Eclipse 80i; Transmission electron microscope, HT7700) after incubating on BHI-1.0% NaCl (w/v) plates at 28°C for 5 days. Oxidase activity was examined by oxidase reagent (bioMérieux) and the catalase activity of strains was tested with bubble production in 3% (v/v) hydrogen peroxide. Gram staining was performed using a Gram-staining kit (Baso) (Austrian, 1960), and motility was tested by observing the spreading growth of cells inoculated by piercing into BHI semisolid (0.3% agar, w/v) medium in test tubes (Xu et al., 2013). Acid production from sucrose, glucose, and other physiological and biochemical tests were determined using API 50CH strips combined with API 50CHB medium and API 20NE, and qualitative enzyme tests were determined with the API ZYM system (bioMérieux). Additionally, Biolog GEN III MicroPlate (catalog No. 1030) with inoculating fluid (catalog No. 72401-IF-A) was also used to obtain the biochemical data following the manufacturers’ instructions (Biolog). Antibiotic sensitivity of strains HY006^T^ and HY1793^T^ was tested using the E-test stripes (bioMérieux) as previously described (Brown and Brown, 1991), on BHI supplemented with 5.0% sheep blood agar at 28°C containing the following antibiotics: ceftriaxone, chloramphenicol, clindamycin, erythromycin, levofloxacin, linezolid, meropenem, tetracycline, and vancomycin.

### Chemotaxonomic characterization

For the biomass of chemotaxonomic assays, all the tested strains were harvested from BHI-1% NaCl plates after 5 days of incubation at 28°C. Cellular fatty acids were extracted, methylated, and identified by using the Sherlock Microbial Identification System (MIDI) according to the manufacturer’s standard protocols (Sasser, 1990). The respiratory isoprenoid quinones were extracted, purified, and analyzed by high performance liquid chromatography (HPLC) (Komagata and Suzuki, 1988) with various menaquinones and ubiquinones (Hu et al., 2001) as references. Polar lipids were analyzed using two-dimensional thin-layer chromatography (2D TLC) after hydrolysis with 6 M HCl at 100°C for 18 hours (Staneck and Roberts, 1974; Harper and Davis, 1979). Peptidoglycan amino acid composition was measured with a Hitachi-8900 high speed amino acid analyzer after hydrolyzing the cell wall, and whole-cell sugars were examined according to the method of Hasegawa et al. (Hasegawa et al., 1983).

Bacterial carotenoid extracts were analyzed at 450 nm using an UPLC system with DAD detector (UPLC, U3000; Thermo Scientific). The analytical conditions were as follows, UPLC: column, YMC Carotenoid S-3 μm (150 × 4.6 mm); column temperature, 40°C; flow rate, 1.0 mL/min; injection volume, 2 μL; solvent system (MeOH: MTBE: H_2_O=20: 75: 5); gradient program, 100:0 v/v at 0 min, 39:61 v/v at 15 min, 0:100 V/V at 25 min, 100:0 v/v at 25.1min, 100:0 v/v at 30 min. Data were acquired on the U3000 UPLC (Thermo Scientific) and processed using chromeleon 7.2 CDS (Thermo Scientific) (Meléndez-Martínez et al., 2010; Irakli et al., 2011). The carotenoid content in samples was calculated as the formulas: Carotenoid content (µg/100 mL) = Read concentration (µg/mL) × Dilution factor × 100.

### Reference strains information

Five reference strains were respectively obtained from the Korean Collection for Type Cultures (*O. cavernae* CFH 30183^T^= KCTC 49018^T^; *O. ciconiae* H23M54^T^= KCTC 49151^T^), Canadian Phytoplankton Culture Collection (*O. flavum* CPCC 203535^T^), the Deutsche Sammlung von Mikroorganismen und Zellkulturen (*O. murale* 01-Gi-040^T^= DSM 22056^T^) and the Guangdong Microbial Culture Collection Center (*O. pratense* W204^T^= GDMCC 1.1391^T^).

## Results

### Phylogenetic and phylogenomic analyses

The 16S rRNA gene sequences of strains HY006^T^/HY008 (1,488 bp) and HY1745/HY1793^T^ (1,510 bp) were obtained. According to the comparison results in the EzBioCloud database, strains HY006^T^ and HY008 were highly similar to those of *Ornithinimicrobium pratense* W204^T^ (99.3%) and *O. flavum* CPCC 203535^T^ (97.3%), whereas strains HY1745 and HY1793^T^ were closest to the type strains *O. ciconiae* H23M54^T^ (98.7%), *O. cavernae* CFH 30183^T^ (98.3%), and *O. murale* 01-Gi-040^T^ (98.1%).

**Figure 1:**
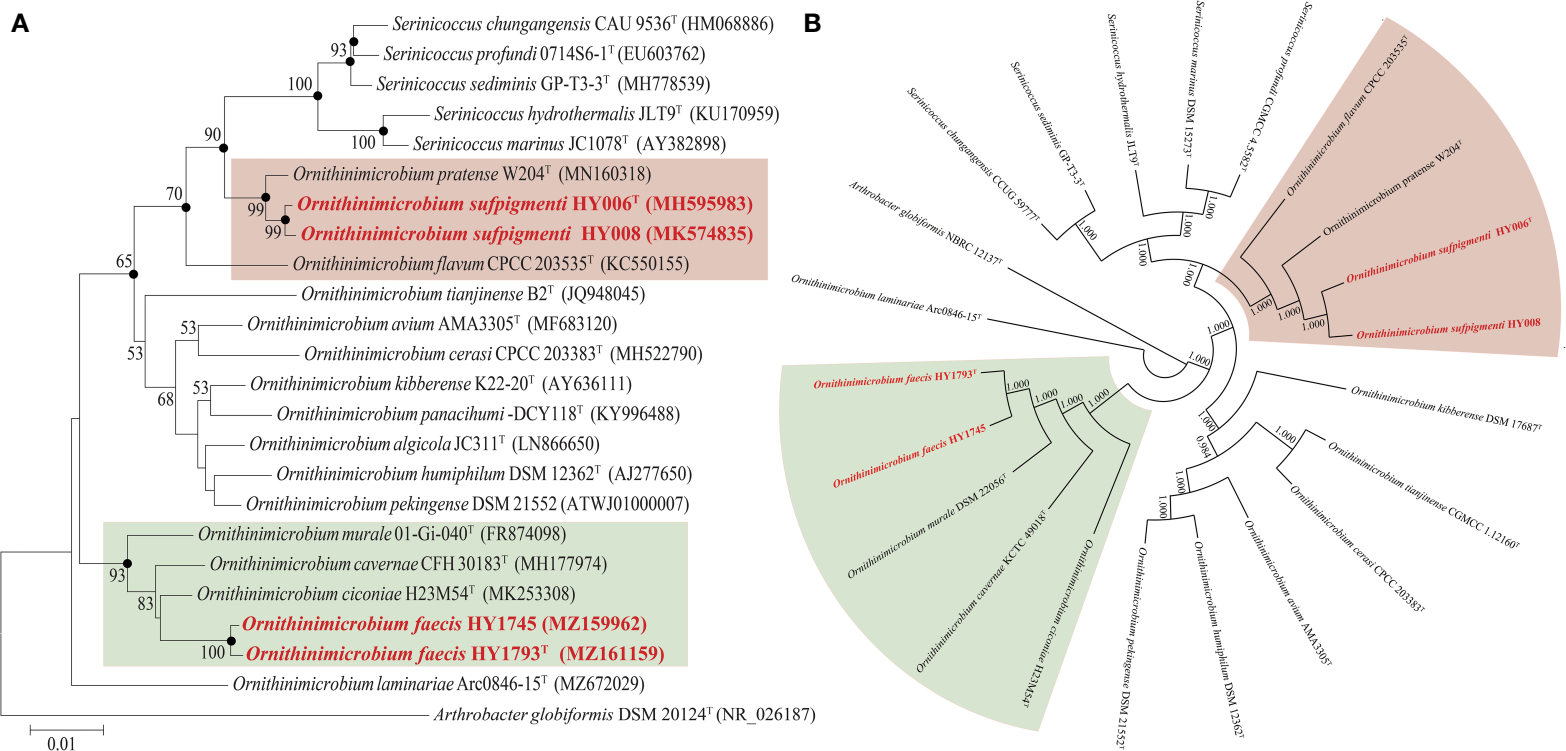
The phylogenetic **(A)** and phylogenomic tree **(B)** of *O. sufpigmenti* sp. nov., *O. faecis* sp. nov. and other species of family *Ornithinimicrobiaceae*. **(A)** Solid circles indicate the nodes supported by maximum-likelihood and maximum-parsimony analyses. Numbers on the tree indicate bootstrap values calculated for 1000 subsets for branch points >50%. Bar, 0.01 substitutions per nucleotide position. **(B)** Numbers on the tree indicate each split in the tree support values with the Shimodaira-Hasegawa test calculated for 1000 resamples.

The phylogenetic trees constructed using various algorithms (NJ, ML, and MP) (**Figure 1A**) showed that strains HY1745 and HY1793^T^ formed a separate phylogenetic sub-branch within a sub-clade in the *Ornithinimicrobium* clade encompassed by three recognized species of the genus (most closely related neighbors mentioned above). In addition, the closest relationship of strains HY006^T^/HY008 to *O. pratense* W204^T^ was clearly shown in **Figure 1A**. Furthermore, the phylogenomic tree (based on 536 core genes) indicated that our four isolates belonged to the genus of *Ornithinimicrobium*, and strains HY006^T^/HY008 formed a distinct cluster with species *O. pratense* W204^T^ and *O. flavum* CPCC 203535^T^. The strains HY1745 and HY1793^T^ also formed a clade with *O. ciconiae* H23M54^T^, *O. cavernae* CFH 30183^T^, and *O. murale* 01-Gi-040^T^ supported by high bootstrap value (**Figure 1B**), almost identical to the results of 16S rRNA gene phylogenetic trees.

### Genome characteristics

The draft genome sequence of strain HY008 consisted of 35 contigs and yielded a genome of 4,147,990 bp (contained 3,817 genes) after assembly. The complete genome of type strain HY006^T^ was 4,169,359 bp (N50: 4,169,359 bp; genome coverage: 240.0×) in length and contained 3,805 genes, with one plasmid (length: 46,843 bp). The plasmid contains 59 coding sequences (CDS) and the G + C content is 66.8 mol%. Most of the protein are hypothetical protein (88.1%), some are enzymes involved in DNA replication (6.8%), two uncharacterized protein (3.4%) and one structural protein (1.7%), no proteins with specific functions were found (**Table S3**). In addition, the DNA G+C content of strains HY006^T^ and HY008 were 71.4 and 71.5 mol%, respectively. Strains HY1745 and HY1793^T^ contained 4,421 and 4,262 genes, respectively. The number of tRNA genes in our four strains was 46, and the G + C content was between 68.7 and 71.5% (**Table 1**). Moreover, stains HY006^T^, HY008, HY1745 and HY1793^T^ contained ten, nine, five, and ten predicted virulence factors, respectively (**Table S4**). Specifically, two virulence factor-like genes, namely *sodA* (superoxide dismutase, VFDB ID: VFG001421) and HSI-3 (type VI secretion system ATPase TssH, VFDB ID: VFG041043) were discovered in most our isolates except for strain HY1745. In addition, five virulence factor-like genes, including those encoding isocitrate lyase *icl* (VFG001381), nitrate reductase subunit alpha *narG* (VFG001391), nitrate reductase subunit beta *narH* (VFG001814), AlgW protein *algW* (VFG014984), and UDP-glucose 6-dehydrogenase *ugd* (VFG048797), were also dominantly found in the type strains HY006^T^ and HY1793^T^, but were absent in strains HY008 and HY1745.

**Table 1:** Differential characteristics between our four isolates and the type strains of closely related species.

Characteristics	1	2	3	4	5	6	7	8	9
Colony color	yellow	yellow	white	white	white	yellow	yellow	beige	yellow
Source of isolation	faeces^#^	faeces*	faeces*	faeces^#^	soil	faeces^&^	leaf	indoor wall	soil
Temperature (°C)	15–40	15–40	15–37	15–37	10–42	15–37	10–37	15–37	15–40
Optimal temperature (°C)	28–30	28–30	28	28	28–30	28–30	28–30	30	30
NaCl range (w/v)	0–7.0	0–7.0	0–9.0	0–9.0	0–7.0	0–7.0	0–5.0	0–11	0–10
Optimal NaCl	1.0	1.0	1.0–1.5	1.0–1.5	1.0–1.5	1.5–2.0	0.5–1.0	1.5–2.0	1.0–2.0
pH range	7.0–11.0	7.0–11.0	6.5–10.0	6.5–10.0	6.5–10.0	7.0–10.0	6.5–9	6.5–9.0	7.0–10.0
Optimal pH	8.0–8.5	8.0–8.5	8.0	8.0	8.0–8.5	8.0	7.0	7.5–8.5	8.0
Total sequence length (bp)	4,169,359	4,147,990	4,664,728	4,620,106	4,558,292	4,092,907	3,712,913	3,285,756	3,539,411
Genome coverage	240.0×	320×	220×	338×	100×	349×	282×	100×	380×
G+C content (%)	71.4	71.5	68.7	68.7	70.9	69.2	72.5	69.2	70.8
N50 (bp)	4,169,359	304,687	261,593	4,620,106	254,396	4,092,907	3,712,913	201,890	3,539,411
rRNA	6	5	5	6	10	6	6	3	6
tRNA	46	46	46	46	49	46	46	63	47
Contigs	1	35	29	1	61	1	1	33	1
Genes	3,805	3,797	4,421	4,262	4,175	3,783	3,584	3,084	3,254
Acid phosphatase	+	+	–	–	–	+	+	+	+
Amidon	–	–	–	–	+	–	–	–	–
D-arabinose	w	–	–	–	+	–	–	w	+
D-fructose	w	–	–	–	w	+	–	w	+
D-galactose	–	–	–	–	w	+	–	+	–
D-glucose	–	–	–	+	+	–	–	+	–
D-maltose	–	–	–	–	+	+	–	–	+
D-raffinose	–	+	–	–	+	–	–	–	w
D-tagatose	w	w	+	+	w	–	–	+	w
Esterase (C4)	+	+	+	+	+	w	+	+	–
L-fucose	–	–	–	–	–	–	–	+	+
α-chymotrypsin	+	+	–	–	–	–	–	+	–
β-glucuronidase	+	+	+	w	–	–	–	–	–

#, *Rousettus leschenaultia*; *, *Taphozous perforates*; &, birds.

Strains: 1, HY006^T^; 2, HY008; 3, HY1745; 4, HY1793^T^; 5, *O. cavernae* KCTC 49018^T^; 6, *O. ciconiae* JCM 33221^T^; 7, *O. flavum* CPCC 203535^T^; 8, *O. murale* DSM 22056^T^; 9, *O. pratense* GDMCC 1.1391^T^. +, positive; w, weakly positive; –, negative.

The values of dDDH between the four isolates and their closely related species or other available genome sequences in the family *Ornithinimicrobiaceae* were all below the 70% threshold (19.6–33.6%), by contrast to the finding that strains HY006^T^ and HY008 had a dDDH value of 99.9% and strains HY1745 and HY1793^T^ shared 78.9%, indicating that they belong to the same species (**Table S2**). Similarly, the ANI values of strain HY006^T^ with strains *O. flavum* CPCC 203535^T^ and *O. pratense* GDMCC 1.1391^T^ were 79.3% and 87.4%, respectively, which were below the threshold value (95%) for species delineation; strain HY1793^T^ with its three closely related species (*O. ciconiae* H23M54^T^, *O. cavernae* KCTC 49018^T^ and *O. murale* DSM22056^T^) were between 80.5–82.9 and ranged from 71.2 to 75.0% with other species in the genus *Ornithinimicrobium.* However, the ANI relatedness within each strain pair is 99.9% (HY006^T^ and HY008) and 97.5% (HY1745 and HY1793^T^). Moreover, the dDDH and ANI values between strains HY006^T^ and HY1793^T^ were 20.4% and 74.4% respectively, suggesting that they belong to different species. In short, these results support the notion that these four strains represent two different novel species in the genus *Ornithinimicrobium*.

**Figure 2:**
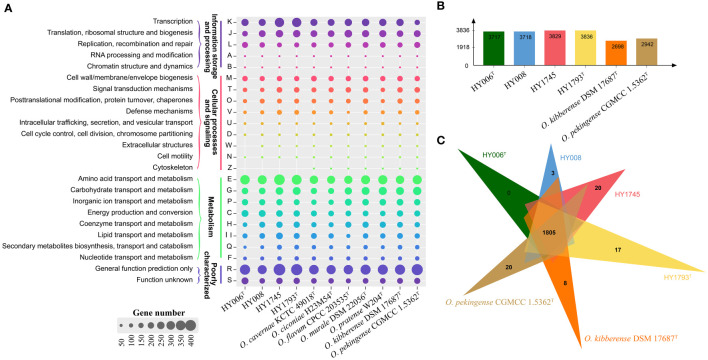
Whole-genome comparison and annotation of orthologous clusters across multiple strains. **(A)** Distributions of the COGs in the eleven sequenced genomes of genus *Ornithinimicrobium*; **(B)** The number of clusters in each strain; **(C)** Venn diagram displays the distribution of shared orthologous clusters among the six strains.

Moreover, the Clusters of Orthologous Groups (COG) database was used for the classification of the genes in the sequenced genomes (**Figure 2A**). The results revealed that the highest numbers of genes contained in these genomes were associated with transcription (COG-K), translation (COG-J), and DNA replication and repair (COG-L) for information storage and processing. For cellular processes and signaling, the genes involved in cell wall/membrane/envelope biogenesis (COG-M) and signal transduction mechanisms (COG-T) were commonly abundant in sequenced genomes. Based on our analysis of the genes associated with metabolism, we found that the species of the genus *Ornithinimicrobium* focused on amino acids transport and metabolism (COG-E) and carbohydrate transport and metabolism (COG-G), additionally, the inorganic ion transport and metabolism (COG-P), energy production and conversion (COG-C) and coenzyme transport and metabolism (COG-H) were also abundant in the bacterial genomes. However, the proportion of secondary metabolites biosynthesis, transport and catabolism (COG-Q) and nucleotide transport and metabolism (COG-F) were relatively small. Notably, a large quantity of the genes in these bacterial genomes were poorly characterized, and their functions remain to be identified (COG-S) or general function prediction only (COG-R) (**Figure 2A**).

Furthermore, the Venn diagram displays the distribution of shared orthologous clusters among our four isolates and the other two strains (*O. kibberense* DSM 17687^T^ and *O. pekingense* CGMCC 1.5362^T^, which should be deserved more attention because they were possessing cytotoxicity activity against to some mammary adenocarcinoma cell lines or causing ocular infection, respectively. In addition, MK-8(H_4_) was detected as the major predominant menaquinone, which was consistent with strains HY006^T^ and HY1793^T^ but different from the other strains isolated from *Rousettus leschenaultia* or *Taphozous perforates*. Furthermore, both of them were color in light yellow, which was also similar to species *O. sufpigmenti*). The six strains form clusters number between 2,698 to 3,836 (**Figure 2B**), and a total of 1805 clusters are shared with the above strains. In addition, there are 3, 20, 17, 8, and 20 single-copy gene clusters in strains HY008, HY1745, HY1793^T^, *O. kibberense* DSM 17687^T^, and *O. pekingense* CGMCC 1.5362^T^, respectively (**Figure 2C**). Additionally, our four strains and the above two strains have large segment sequence rearrangement. There are at least five locally collinear blocks, and the direction and arrange sequence are consistent, suggesting that genome evolution may experience a similar path (**Figure S1**).

### Phenotypic, physiological and chemotaxonomic characteristics

Collectively, cells of strains HY006^T^, HY008, HY1745, and HY1793^T^ were convex, opaque, and circular (0.5–1.0 mm in diameter) with smooth surfaces. Cells were Gram-stain-positive, catalase-positive, oxidase-negative, circle-shaped (HY006^T^ 1.0 × 1.2 µm; HY1793^T^ 1.0 × 1.2 µm) (**Figure S2**), non-motile. Interestingly, the color was totally different between the two type strains. The strain HY006^T^ was yellow whereas strain HY1793^T^ was cream, which may be associated to the production of carotenoids. Extraction of the HY006^T^ cells with a solvent system showed that they accumulated two different carotenoids at significant concentrations (**Figure S3**). The contents of β-cryptoxanthin (C_40_H_56_O) and lycopene (C_40_H_56_) in strain HY006^T^ were 26.0 µg/mL and 2.3 µg/mL, respectively. In the contrast, any carotenoid components were not found in the strain of HY1793^T^, which was consistent with its color (cream) but different from strain HY006^T^ (yellow).

Optimal growth conditions for the strains HY006^T^ and HY008 were 28–30°C (range, 15–40°C) for 5 days on BHI with 1.0% (w/v) NaCl [range, 0–7.0 (w/v)] at pH 8.0–8.5 (range, 7.0–11.0). Additionally, the optimal growth conditions of strains HY1745 and HY1793^T^ were at 28°C (range, 15–37°C), pH 8.0 (range, pH 6.5–10.0), and with 1.0–1.5% (w/v) NaCl [up to 9.0% (w/v)] in aerobic. Notably, our strains were tolerant to 7.0% NaCl, while no growth of *O. flavum* CPCC 203535^T^ was observed under this condition. However, *O. cavernae* KCTC 49018^T^, *O. ciconiae* JCM 33221^T^, *O. murale* DSM 22056^T^, and *O. pratense* GDMCC 1.1391^T^ can grow well with 7.0% NaCl. The four isolates (HY006^T^, HY008, HY1745 and HY1793^T^) and their closely related strains (*O. cavernae* KCTC 49018^T^, *O. ciconiae* JCM 33221^T^, *O. flavum* CPCC 203535^T^, *O. murale* DSM 22056^T^ and *O. pratense* GDMCC 1.1391^T^) were also not observed growth on pH value of lower than 6.0, which were different from strains *O. pekingense* DSM 21552^T^ and *O. kibberense* DSM 17687^T^ but consistent with most the species of *Ornithinimicrobium* (Huo et al., 2019; Fang et al., 2020; Cao et al., 2022). Also, strains HY006^T^ and HY008 were positive for α-chymotrypsin, which were different from strains HY1745, HY1793^T^, *O. cavernae* KCTC 49018^T^, *O. ciconiae* JCM 33221^T^, *O. flavum* CPCC 203535^T^, *O. pratense* GDMCC 1.1391^T^ but consistent with *O. murale* DSM 22056^T^. Conversely, alkaline phosphatase and cystine arylamidase were utilized by strains HY1745 and HY1793^T^ whereas strains HY006^T^ and HY008 were negative for them. The more differential biochemical characteristics of all the compared strains were shown in **Table S5**. As detailed in **Figure S4**, all strains positively assimilated aztreonam, d-serine and lithium chloride while tested with the Biolog GEN III MicroPlate. Of note is the strains HY006^T^ and HY008 were positive for acetoacetic acid, d-arabitol, d-glucuronic acid, l-galactonic acid lactone, tetrazolium violet and α-hydroxy-butyric acid, distinctly different from the other two potential novel strains (HY1745 and HY1793^T^). Most notably, lincomycin was found to be utilized by strains HY1745 and HY1793^T^, which was consistent with strains *O. cavernae* CFH 30183^T^, *O. murale* DSM 22056^T^, *O. kibberense* K22-20^T^ and *O. panacihumi* DCY118^T^ (Mayilraj et al., 2006; Huo et al., 2019; Zhang et al., 2019). Conversely, strains HY006^T^, HY008 and the other four closely related strains (*O. ciconiae* JCM 33221^T^, *O. flavum* CPCC 203535^T^, and *O. pratense* GDMCC 1.1391^T^) were sensitive to lincomycin according to the results of Biolog GEN III MicroPlate (**Figure S4**). Furthermore, strain HY006^T^ was resistant [minimum inhibitory concentration (MIC) ≥8.0 µg/mL] to chloramphenicol (MIC, >20.0 µg/mL), linezolid (MIC, 12 µg/mL), and intermediately resistant (MIC, 1.0–6.0 µg/mL) to erythromycin (MIC, 3.0 µg/mL) and tetracycline (MIC, 6.0 µg/mL), but susceptible to ceftriaxone, clindamycin levofloxacin, meropenem and vancomycin (MIC, ≤0.5 µg/mL). Additionally, strain HY1793^T^ was resistant to erythromycin (MIC, 12.0 µg/mL), intermediately resistant to clindamycin (MIC, 2.0–3.0 µg/mL) and levofloxacin (MIC, 1.0 µg/mL) about the antibiotic sensitivity whereas susceptible to ceftriaxone, chloramphenicol, linezolid, meropenem, tetracycline and vancomycin (MIC, ≤0.5 µg/mL) (**Figure S5**). Of note, both strains contain the corresponding drug resistance genes in their genome but the value of identities is low (<60.0%), indicating that the resistant phenotype may be regulated by other different genes and will require more research to be identified.

The major cellular fatty acids (>20.0%) of our four isolates (HY006^T^, HY008, HY1745 and HY1793^T^) were *iso*-C_15:0_ and *iso*-C_16:0_, which were consistent with strain *O. humiphilum* HKI 0124^T^ (Groth et al., 2001) and *O. murale* 01-Gi-040^T^ (Kampfer et al., 2013). In addition, Summed Feature 9 (C_16:0_10-methyl and/or *iso*-C_17:1_ *ω*9*_C_*) was also accounted for a high proportion (>10.0%) in strains HY1745, HY1793^T^ and other closely related strains (*O. cavernae* KCTC 49018^T^, *O. ciconiae* JCM 33221^T^, *O. flavum* CPCC 203535^T^, *O. murale* DSM 22056^T^, and *O. pratense* GDMCC 1.1391^T^) but lower in strains HY006^T^ and HY008 (<6.5%). The detailed fatty acid profiles of the strains are presented in **Table S6**. MK-8(H_4_) was detected as the predominant menaquinone in strains HY006^T^ and HY1793^T^, which was accounted for 59.0% and 93.3%, respectively. Furthermore, a significant amount of MK-8 (33.9%) was present in strain HY006^T^ rather than MK-6 (6.7%) was shown in strain HY1793^T^, different from other species that had either simpler or more complicated compositions. For example, *O. pekingense* LW6^T^ has been reported to contain partially saturated menaquinone MK-8(H_2_) (Liu et al., 2008); *O. cerasi* CPCC 203383^T^ and *O. flavum* CPCC 203535^T^ had MK-8(H_4_), MK-8(H_2_), and MK-8 as the predominant quinone (Fang et al., 2017; Fang et al., 2020). Both type strains (HY006^T^ and HY1793^T^) had diphosphatidylglycerol (DPG), phosphatidylglycerol (PG), phosphatidyl inositol mannoside (PIM), two unidentified lipids (L1-2) and two unknown phosphoglycolipids (PGL1-2) in their polar lipid profiles (**Figure S6**), additionally, HY006^T^ had two phospholipids (PL1-2) and two unidentified glycolipids (GL1-2) whereas HY1793^T^ had five phospholipids (PL1-5) and five unidentified glycolipids (GL1-5). The whole-cell peptidoglycan of both type strains contained ornithine, alanine, glycine, and glutamic acid (**Figure S7A**), significantly, ornithine is the diagnostic diamino acid component within the cell wall of the genus *Ornithinimicrobium*. They also contained glucose in the whole cell sugar, however, ribose was only detected in strain HY1793^T^ (**Figure S7B**).

## Discussion

Compared with viruses, there are few studies on the microorganism carried by bats, particularly the reports of new bacterial species that are straightforward to be neglected (Muhldorfer, 2013). Our laboratory has investigated the intestinal and fecal flora of bats since 2018 to identify potentially pathogenic microbial species and dissect the process of disease transmission (Huang et al., 2022c). So far, we have published over ten new species of bacteria isolated from bat feces, among which *Brevibacterium zhoupengii* (Huang et al., 2022a), *Microbacterium fandaimingii* (Zhou et al., 2021), *Gordonia zhenghanii*, *G. liuliyuniae* (Huang et al., 2022b), *Tomitella gaofuii* and *T. fengzijianii* (Huang et al., 2022d) belong to the phylum Actinobacteria. As previously described, actinomycete bacteria are highly prevalent in *Rousettus leschenaultia* and *Taphozous perforates* through traditional isolation methods. Though *O. sufpigmenti* and *O. faecis* belongs to Actinobacteria, it differ from other actinobacterial strains isolated from *R. leschenaultia* and *T. perforates* by the presence of MK-8(H_4_) as major menaquinone. In addition, the optimal temperature of actinomycetes isolated from the faeces of *Rousettus leschenaultia* and/or *Taphozous perforates* is about 28°C, different from the strain *Apibacter raozihei* HY039 (belonged to Bacteroidota) best growth in 35°C. Furthermore, most of them are Gram-stain-positive, have high G+C content, and are creamy in color. However, *Ornithinimicrobium sufpigmenti* and *O. faecis* were observed to grow on the plates higher than 37°C, which was distinct from other actinomycetes but similarity to *Apibacter raozihei* (**Table S7**).

Moreover, the fecal microbiota in bats was found to be diverse with predominant bacterial phylum Proteobacteria and Firmicutes (Sun et al., 2020; Huang et al., 2022c). The presence of opportunistic pathogens *Citrobacter freundii*, *Escherichia coli*, *Enterococcus faecalis*, *Serratia fonticola, Shigella flexneri*, and *Rahnella aquatilis* were also recorded (Muhldorfer, 2013; Wolkers-Rooijackers et al., 2019). However, there is no proof that bats are a major vector for the transmission of bacterial zoonotic diseases (Wolkers-Rooijackers et al., 2019). Interestingly, *Ornithinimicrobium pekingense* has been reported to cause ocular infection by Borsali (Borsali et al., 2011). There are nine and ten predicted virulence factors in strains *O. kibberense* DSM 17687^T^ and *O. pekingense* CGMCC 1.5362^T^, respectively (**Table S4**). Specifically, iron-dependent repressor and activator *ideR* (VFDB ID: VFG001406) was dominantly found in these two strains whereas absent in our four isolates. However, no specific virulence factor related genes or molecular mechanism can explain their potential pathogenicity according to searching against the virulence factor database (http://www.mgc.ac.cn/VFs/) and annotating by the RAST server. Two virulence factor-like genes, namely *sodA* (superoxide dismutase, VFDB ID: VFG001421) and HSI-3 (type VI secretion system ATPase TssH, VFDB ID: VFG041043) were discovered among all the compared strains except for strain HY1745. Interestingly, superoxide dismutase is important for *Mycobacterium tuberculosis* survival during infection (Edwards et al., 2001; Smith, 2003). The type VI secretion system (T6SS) participates in interbacterial competition as well as pathogenesis, it is a multiprotein complex widespread in Proteobacteria and dedicated to the delivery of toxins into both prokaryotic and eukaryotic cells, such as the type VI secretion system of *Pseudomonas aeruginosa* targets a toxin to bacteria (Hood et al., 2010; Journet and Cascales, 2016). Therefore, more research is needed to deepen the understanding of the genus *Ornithinimicrobium.* About the existence of *Ornithinimicrobium* sp. within these bats, we considered that these *Ornithinimicrobium* spp. might be a passing flora, probably not a long-term colonizer in the bat gut.

## Conclusion

Based on evidence from the phylogenetic, genomic, physiological, biochemical, as well as chemotaxonomic findings suggest that our four strains represent two novel species of the genus *Ornithinimicrobium*, for which the names *Ornithinimicrobium sufpigmenti* (suf.pig.men’ti, N.L. neut. adj. *sufpigmenti*, referring to the production of pigment of β-cryptoxanthin and lycopene) sp. nov. (HY006^T^ and HY008) and *Ornithinimicrobiumfaecis* (faeˊcis. L. gen. fem. n. *faecis* of faeces, as the organism was found in bat faeces) sp. nov. (HY1745 and HY1793^T^) are proposed, respectively. The type strains are HY006^T^ (=CGMCC 1.16565^T^ =JCM 33397^T^) and HY1793^T^ (=CGMCC 1.19143^T^ =JCM 34881^T^), respectively, were isolated from the feces of *Rousettus leschenaultia* in 2018, which were collected at Chuxiong Yi Autonomous Prefecture of Yunnan Province in 2013, China.

## Data availability statement

The datasets presented in this study can be found in online repositories. The names of the repository/repositories and accession number(s) can be found in the article/**Supplementary Material**.

## Ethics statement

The ethical practice was approved by the Ethical Committee of the National Institute for Communicable Disease Control and Prevention, Chinese Center for Disease Control and Prevention (NO: ICDC-2016004).

## Author contributions

JX and JJ conceived the study. YH, SZ, YT, WL, SL and DJ performed the sampling, isolating and sequencing. YH, JY, JP, JJ and JX analyzed the data and drafted the manuscript. HZ and LL supervised the data analysis. All authors contributed to the article and approved the submitted version.
