# Supplementary material for: Morphological and genomic characteristics of two novel actinomycetes, Ornithinimicrobium sufpigmenti sp. nov. and Ornithinimicrobium faecis sp. nov. isolated from bat faeces (Rousettus leschenaultia and Taphozous perforates)
Source: Front Cell Infect Microbiol. 2023 Feb 14;13:1093407. doi: 10.3389/fcimb.2023.1093407 (PMC9973731; doi:10.3389/fcimb.2023.1093407)
Supplement: Supplementary file 1 [file DataSheet_1.pdf]

Morphological and genomic characteristics of two novel actinomycetes, *Ornithinimicrobium sufpigmenti* sp. nov. and *Ornithinimicrobium faecis* sp. nov. isolated from bat faeces (*Rousettus leschenaultia* and *Taphozous perforates*)

Yuyuan Huang[1], Suping Zhang[1,2], Yuanmeihui Tao[1], Jing Yang[1,3], Shan Lu[1,3], Dong Jin[1,3], Ji Pu[1], Wenbo Luo[1], Han Zheng[1], Liyun Liu[1], Jia-fu Jiang[4]* and Jianguo Xu[1,2,3,5]*

1 State Key Laboratory of Infectious Disease Prevention and Control, National Institute for Communicable Disease Control and Prevention, Chinese Center for Disease Control and Prevention, Beijing 102206, PR China.

2 Department of Epidemiology, Center for Global Health, School of Public Health, Nanjing Medical University, Nanjing 211166, Jiangsu Province, PR China.

3 Research Units of Discovery of Unknown Bacteria and Function, Chinese Academy of Medical Sciences, Beijing 100730, PR China.

4 Beijing Institute of Microbiology and Epidemiology, State Key Laboratory of Pathogen and Biosecurity, Beijing, 100071, PR China.

5 Research Institute of Public Health, Nankai University, Tianjin 300350, PR China.

***Correspondence:** Jianguo Xu, State Key Laboratory of Infectious Disease Prevention and Control, National Institute for Communicable Disease Control and Prevention, Chinese Center for Disease Control and Prevention, Changping, Beijing 102206, PR China. Email: xujianguo@icdc.cn;

Jiafu Jiang, Beijing Institute of Microbiology and Epidemiology, State Key Laboratory of Pathogen and Biosecurity, Beijing, 100071, China. Email: jiangjf2008@139.com.

Table S1. Sample collection locations and bat community.

Genus	Feeding habit	Collecting time	Collecting location (Latitude and longitude)
Rousettus spp.	Frugivorous	October 23, 2013	Chuxiong Yi Autonomous Prefecture, Yunnan Province (N25°09′10″, E102°04′39″)
Taphozous spp.	Insectivorous	July 28, 2011	Chongzuo City, Guangxi Province (N22°20′54″, E106°49′20″)

Table S2. Digital DDH values between our four isolates and the type strains in family *Ornithinimicrobiaceae.*

Strains: 1, HY006^T; 2, HY008; 3, HY1745; 4, HY1793^T.

Strains	Accession number	dDDH values (%)				ANI values (%)			
		1	2	3	4	1	2	3	4
Arthrobacter globiformis NBRC 12137^T	BAEG00000000	18.8	18.4	19.5	19.6	70.1	69.8	69.6	69.7
Ornithinimicrobium avium AMA3305^T	CP031229	21.8	21.8	20.3	20.4	78.1	78.2	74.9	75.0
O. cavernae KCTC 49018^T	QDDJ00000000	20.6	20.5	24.6	24.8	74.7	74.8	81.4	81.4
O. cerasi CPCC 203383^T	QZMN00000000	21.6	21.6	19.8	20.1	77.9	77.9	74.4	74.2
O. ciconiae H23M54^T	CP041616	21.0	20.9	24.0	23.8	74.8	74.6	80.5	80.5
O. flavum CPCC 203535^T	CP038213	22.5	22.5	20.0	20.3	79.3	79.2	74.6	74.3
O. humiphilum DSM 12362^T	VFPU00000000	21.7	21.7	20.0	20.0	78.2	78.1	74.7	74.6
O. kibberense DSM 17687^T	VFXM00000000	21.8	21.8	20.3	20.5	77.9	78.3	74.6	74.6
O. laminariae Arc0846-15^T	JAHZSU000000000	19.8	19.8	19.6	19.7	71.0	70.6	71.2	71.2
O. murale DSM 22056^T	QLVD00000000	20.6	20.6	26.5	26.3	74.9	74.8	83.0	82.9
O. pekingense DSM 21552^T	ATWJ00000000	21.9	21.8	20.0	20.3	78.4	78.3	74.6	74.4
O. pratense W204^T	CP044427	33.7	33.6	19.8	19.9	87.4	87.4	73.9	74.0
O. tianjinense CGMCC 1.12160^T	BMEM00000000	21.5	21.5	20.2	20.3	77.8	77.6	74.4	74.4
Serinicoccus marinus DSM 15273^T	CP043808	21.4	21.3	20.1	19.9	77.5	77.7	74.4	74.0
S. chungangensis CCUG 59777^T	CP040887	21.2	21.1	19.9	20.2	77.4	77.4	73.8	73.7
S. hydrothermalis JLT9^T	CP014989	21.6	21.5	20.2	20.0	78.1	77.8	74.2	74.0
S. profundi CGMCC 4.5582^T	CP042862	21.4	21.3	20.1	19.9	77.5	77.7	74.1	74.0
S. sediminis GP-T3-3^T	SDVA00000000	21.3	21.3	20.0	19.8	77.7	77.8	73.9	74.1
***O. sufpigmenti* HY006^T**	CP036403	-	99.9	20.5	20.4	-	99.9	74.2	74.4
***O. sufpigmenti* HY008**	SKBS00000000	99.9	-	20.4	20.4	99.9	-	74.2	74.4
***O. faecis* HY1745**	JALKVI000000000	20.5	20.4	-	78.9	74.2	74.2	-	97.5
***O. faecis* HY1793^T**	CP099489	20.4	20.4	78.9	-	74.4	74.4	97.5	-

Table S3. Predicted protein list from plasmid in strain HY006^T

Name	Start	End	Length (bp)	Protein Name
HY006_plasmid_0001	124	360	236	Uncharacterized HTH-type transcriptional regulator YvzC
HY006_plasmid_0002	357	809	452	Hypothetical protein
HY006_plasmid_0003	806	949	143	Hypothetical protein
HY006_plasmid_0004	1030	1353	323	Hypothetical protein
HY006_plasmid_0005	1350	1664	314	Hypothetical protein
HY006_plasmid_0006	1664	1846	182	Hypothetical protein
HY006_plasmid_0007	1843	2784	941	Hypothetical protein
HY006_plasmid_0008	2788	3963	1175	Hypothetical protein
HY006_plasmid_0009	3960	4715	755	DNA polymerase III PolC-type
HY006_plasmid_0010	4712	4918	206	Hypothetical protein
HY006_plasmid_0011	4960	5451	491	Hypothetical protein
HY006_plasmid_0012	5476	5796	320	Hypothetical protein
HY006_plasmid_0013	5793	6026	233	Hypothetical protein
HY006_plasmid_0014	6026	7087	1061	Hypothetical protein
HY006_plasmid_0015	7106	7684	578	Single-stranded DNA-binding protein
HY006_plasmid_0016	7711	8088	377	Hypothetical protein
HY006_plasmid_0017	8081	8242	161	Hypothetical protein
HY006_plasmid_0018	8246	9097	851	Hypothetical protein
HY006_plasmid_0019	9225	9917	692	Hypothetical protein
HY006_plasmid_0020	9914	11299	1385	Replicative DNA helicase
HY006_plasmid_0021	11303	11848	545	Hypothetical protein
HY006_plasmid_0022	11845	12207	362	Hypothetical protein
HY006_plasmid_0023	12204	12398	194	Hypothetical protein
HY006_plasmid_0024	12395	12667	272	Hypothetical protein
HY006_plasmid_0025	12657	12899	242	Hypothetical protein
HY006_plasmid_0026	12896	13657	761	Hypothetical protein
HY006_plasmid_0027	13869	14381	512	Hypothetical protein
HY006_plasmid_0028	14378	15682	1304	Hypothetical protein
HY006_plasmid_0029	15669	17201	1532	Hypothetical protein
HY006_plasmid_0030	17229	18332	1103	Structural protein
HY006_plasmid_0031	18316	18489	173	Hypothetical protein
HY006_plasmid_0032	18537	19250	713	Hypothetical protein
HY006_plasmid_0033	19290	20243	953	Hypothetical protein
HY006_plasmid_0034	20301	20486	185	Hypothetical protein
HY006_plasmid_0035	20503	20928	425	Hypothetical protein
HY006_plasmid_0036	20925	21227	302	Hypothetical protein
HY006_plasmid_0037	21390	21866	476	Hypothetical protein
HY006_plasmid_0038	21870	22211	341	Hypothetical protein
HY006_plasmid_0039	22224	22568	344	Hypothetical protein
HY006_plasmid_0040	22577	22918	341	Hypothetical protein
HY006_plasmid_0041	22902	23345	443	Hypothetical protein
HY006_plasmid_0042	23422	23892	470	Hypothetical protein
HY006_plasmid_0043	24002	24469	467	Hypothetical protein
HY006_plasmid_0044	24680	27496	2816	Uncharacterized protein Yqb
HY006_plasmid_0045	27493	28326	833	Hypothetical protein

HY006_plasmid_0046	28377	32249	3872	Hypothetical protein
HY006_plasmid_0047	32246	32413	167	Hypothetical protein
HY006_plasmid_0048	32410	33609	1199	Hypothetical protein
HY006_plasmid_0049	33685	34122	437	Hypothetical protein
HY006_plasmid_0050	34125	34538	413	Hypothetical protein
HY006_plasmid_0051	34585	35829	1244	Hypothetical protein
HY006_plasmid_0052	35836	36117	281	Hypothetical protein
HY006_plasmid_0053	36279	36437	158	Hypothetical protein
HY006_plasmid_0054	36443	36535	92	Hypothetical protein
HY006_plasmid_0055	36668	38119	1451	Putative DNA recombinase
HY006_plasmid_0056	38103	38345	242	Hypothetical protein
HY006_plasmid_0057	38336	38767	431	Hypothetical protein
HY006_plasmid_0058	39210	39482	272	Hypothetical protein
HY006_plasmid_0059	39490	39999	509	Hypothetical protein

Table S4. The virulence factor genes of *Ornithinimicrobium sufpigmenti*, *O. faecis, O. kibberense* and *O. pekingense*

VFDB IDs are based on the Virulence Factor Database (http://www.mgc.ac.cn/VFs/main.htm). Strains: 1, HY006^{T}; 2, HY008;3, HY1745; 4, HY1793^{T}; 5, DSM 17687^{T}; 6, CGMCC 1.5362^{T}. Symbol: +, presence; –, absence.

VFDB ID	Description	Related VF	Origin of the virulence factor	1	2	3	4	5	6	Identities	Score (bits)
**VFG001421**	(sodA) superoxide dismutase	SodA	*Mycobacterium tuberculosis* H37Rv	+	+	-	+	+	+	87-95%	82-103
**VFG041043**	(tssH) type VI secretion system ATPase TssH	HSI-3	*Pseudomonas aeruginosa* PAO1	+	+	-	+	+	+	87-88%	84-90
**VFG001381**	(icl) Isocitrate lyase Icl (isocitrase)	Isocitrate lyase	*Mycobacterium tuberculosis* H37Rv	+	-	-	+	-	-	86-87%	151-182
**VFG001391**	(narG) nitrate reductase subunit alpha	Nitrate reductase	*Mycobacterium tuberculosis* H37Rv	+	-	-	+	-	-	82-84%	103-149
**VFG001405**	(sigA/rpoV) RNA polymerase sigma factor SigA	SigA	*Mycobacterium tuberculosis* H37Rv	+	-	+	+	-	-	83-89%	331-474
**VFG001814**	(narH) nitrate reductase subunit beta	Nitrate reductase	*Mycobacterium tuberculosis* H37Rv	+	-	-	+	-	-	83-84%	218-236
**VFG014984**	(algW) AlgW protein	Alginate	*Pseudomonas aeruginosa* PAO1	+	-	-	+	-	-	100%	72-86
**VFG048797**	(ugd) UDP-glucose 6-dehydrogenase	Capsule	*Klebsiella pneumoniae subsp. pneumoniae* NTUH-K2044	+	-	-	+	-	-	79-83%	86-151
**VFG000035**	(bplD) UDP-N-acetylglucosamine 2-epimerase	LPS	*Bordetella pertussis* Tohama I	+	-	-	-	-	-	84%	94
**VFG015515**	(phzE1) phenazine biosynthesis protein PhzE	Pyocyanin	*Pseudomonas aeruginosa* PAO1	+	-	-	-	-	-	83%	84
**VFG002480**	(tssH-5/clpV) Clp-type ATPase chaperone protein	T6SS-1	*Burkholderia pseudomallei* K96243	-	+	-	+	+	+	83-85%	74-88
**VFG016041**	(pvdI) peptide synthase	Pyoverdine	*Pseudomonas aeruginosa* PAO1	-	-	-	+	-	-	95%	72
**VFG001406**	(ideR) Iron-dependent repressor and activator	IdeR	*Mycobacterium tuberculosis* H37Rv	-	-	-	-	+	+	89-96%	54-56
**VFG002076**	(clpV1) type VI secretion system AAA+ family	HSI-1	*Pseudomonas aeruginosa* PAO1	-	+	-	-	+	+	84-85%	78-90
**VFG038395**	(clpB) type VI secretion system ATPase ClpV1	T6SS	*Aeromonas hydrophila subsp. hydrophila* ATCC 7966	-	+	-	-	+	+	82-95%	64-72
**VFG041020**	(tssH) type VI secretion system ATPase TssH	HSI-2	*Pseudomonas aeruginosa* PAO1	-	+	-	-	+	+	83-91%	54-76
**VFG001379**	(hspR) heat shock protein transcriptional repressor HspR	HspR	*Mycobacterium tuberculosis* H37Rv	-	-	-	-	-	+	89%	58
**VFG048697**	(clpV/tssH) type VI secretion system ATPase TssH	T6SS	*Klebsiella pneumoniae subsp. pneumoniae* NTUH-K2044	-	+	-	-	-	+	85-90%	50-56
**VFG001266**	(pchI) ABC transporter ATP-binding protein	Pyochelin	*Pseudomonas aeruginosa* PAO1	-	-	-	-	+	-	94%	52
**VFG001269**	(cyaB) cyclolysin secretion ATP-binding protein	Cya	*Bordetella pertussis* Tohama I	-	-	-	-	+	-	90%	54
**VFG007904**	(ddrA) daunorubicin ABC transporter ATP-binding protein DrrA	PDIM	*Mycobacterium tuberculosis* H37Rv	-	+	-	-	+	-	94%	52-60
**VFG000477**	(rpoS) RNA polymerase sigma factor RpoS	RpoS	*Salmonella enterica subsp. enterica serovar* Typhimurium str. LT2	-	-	+	-	-	-	88%	58
**VFG002059**	(tagT) type VI secretion associated protein TagT	HSI-1	*Pseudomonas aeruginosa* PAO1	-	+	-	-	-		94%	54
**VFG038533**	(flpF) traffic ATPase	Flp type IV pili	*Aeromonas hydrophila* ML09-119	-	-	+	-	-	-	90%	50
**VFG043634**	(VV1_RS15610) CpaF family protein	Flp pili	*Vibrio vulnificus* CMCP6	-	-	+	-	-	-	85%	46
**VFG043648**	(rpoS) RNA polymerase sigma factor RpoS	Type IV pili	*Pseudomonas aeruginosa* PAO1	-	-	+	-	-	-	96%	52

Table S5. The biochemical characteristics of *O. sufpigmenti* sp. nov., *O. faecis* sp. nov. and the type strains of closely related species

Strains: 1, HY006^{T}; 2, HY008; 3, HY1745; 4, HY1793^{T}; 5, *O. cavernae* KCTC 49018^{T};6, *O. ciconiae* JCM 33221^{T}; 7, *O. flavum* CPCC 203535^{T}; 8, *O. murale* DSM 22056^{T}; 9, *O. pratense* GDMCC 1.1391^{T}. +, positive; w, weakly positive; –, negative.

Characteristics	1	2	3	4	5	6	7	8	9
**API ZYM**									
Alkaline phosphatase	–	–	+	–	–	–	–	–	–
Esterase Lipase (C8)	+	+	w	w	+	+	w	–	+
Lipase (C14)	–	–	–	–	–	–	–	–	–
Leucine arylamidase	+	+	+	+	+	+	+	–	+
Valine arylamidase	–	–	–	–	–	–	–	–	–
Cystine arylamidase	w/–	w/–	+	+	–	–	–	w	+
Trypsin	w	w	–	–	–	–	+	w	–
Naphthol-AS-BI-phosphophdrolase	+	+	+	+	+	+	+	–	+
α-galactosidase	–	w	–	–	–	–	–	–	–
β-galactosidase	–	–	–	–	–	–	w	+	+
α-glucosidase	+	+	+	+	+	+	+	–	+
β-glucosidase	–	–	–	–	–	–	–	+	–
**API 50CH**									
glycerol	–	–	–	–	–	w	w	–	–
erythritol	–	–	–	–	–	–	–	–	–
L-arabinose	–	–	–	–	–	–	+	–	–
D-ribose	w	w	+	+	+	+	+	–	+
D-xylose	w	w	–	–	–	w	–	–	–
L-xylose	–	–	–	–	–	–	–	–	–
D-adonitol	–	+	–	–	–	–	–	–	–
Methyl-βD-xylopyranoside	–	–	–	–	–	–	–	–	–
D-mannose	–	–	–	–	–	–	+	–	–
L-sorbose	–	–	–	w	–	w	–	–	–
inositol	+	–	–	–	–	–	–	–	–
D-mannitol	–	–	–	–	–	–	–	w	+
D-sorbitol	–	w	–	–	–	–	+	–	–
arbutin	w	+	–	–	–	w	–	–	–
esculin ferric citrate	+	+	+	+	+	+	+	–	+
salicin	–	–	–	–	–	–	–	–	–
D-cellobiose	–	–	–	–	–	w	w	–	w
D-lactose	–	–	–	–	–	–	–	–	–
D-melibiose	–	–	–	–	–	–	+	+	+
D-saccharose	w	+	–	–	w	+	+	–	w
D-trehalose	w	–	–	–	w	+	+	–	w
glycogen	–	–	–	–	w	+	+	–	–
gentiobiose	–	–	–	–	–	–	w	+	w
D-turanose	–	–	–	–	–	+	w	–	w
D-lyxose	–	–	–	w	–	–	–	–	–

D-fucose	–	–	–	–	–	–	+	–	–
D-arabitol	w	w	–	–	–	–	–	–	–
L-arabitol	–	–	–	–	–	–	–	–	–
potassium gluconate	–	–	–	–	–	–	–	–	–
potassium 2-ketogluconate	–	–	–	–	–	–	–	–	–
potassium 5-ketogluconate	+	+	+	+	+	+	+	–	+
**API 20NE**									
Potassium nitrate	+	+	w	–	+	+	+	–	+
L-tryptophane	–	–	–	–	–	–	–	–	–
D-glucose	–	–	–	–	–	–	+	–	+
L-arginine	–	–	–	–	–	–	+	–	–
urea	–	–	–	–	–	–	+	+	–
Esculin Ferric citrate (aesculin)	+	+	+	+	+	+	+	–	+
gelatin	+	+	+	+	+	+	+	–	+
capric acid	–	–	–	–	–	–	–	–	–
adipic acid	–	+	–	–	–	–	+	w	+
malic acid	–	–	–	–	–	–	+	–	+
trisodium citrate	–	–	–	–	–	–	+	w	+
phenylacetic acid	–	–	–	–	–	–	–	w	–

Table S6. Cellular fatty acid contents (%) of *O. sufpigmenti* sp. nov., *O. faecis* sp. nov. and the type strains of closely related species

Strains: 1, HY006^T; 2, HY008; 3, HY1745; 4, HY1793^T; 5, *O. cavernae* KCTC 49018^T;6, *O. ciconiae* JCM 33221^T; 7, *O. flavum* CPCC 203535^T; 8, *O. murale* DSM 22056^T; 9, *O. pratense* GDMCC 1.1391^T. Percentages of total fatty acids were determined in this study, with bold face indicating major fatty acids (>10%). ND, not detected.

**Fatty acids (%)**	**1**	**2**	**3**	**4**	**5**	**6**	**7**	**8**	**9**
$C_{14:0}$	0.2	0.1	0.1	0.3	ND	0.1	0.9	0.2	0.7
$C_{15:1}\omega6c$	ND	ND	0.2	ND	0.5	ND	ND	ND	ND
$C_{15:1}\omega8c$	ND	ND	0.3	ND	0.2	ND	ND	ND	ND
$C_{16:0}$	1.1	1.0	1.2	2.3	1.1	4.1	9.8	2.0	8.3
$C_{16:1}\omega9c$	ND	ND	0.2	0.2	ND	ND	ND	0.3	0.4
$C_{17:0}$ 3OH	0.2	0.2	0.2	0.2	0.2	0.2	ND	0.3	ND
$C_{17:1}\omega5c$	0.1	0.3	ND	0.2	ND	ND	ND	ND	ND
$C_{17:1}\omega6c$	ND	ND	0.3	0.4	0.2	ND	ND	ND	ND
$C_{17:1}\omega8c$	ND	ND	0.7	1.1	ND	0.2	ND	ND	ND
$C_{18:0}$	0.3	0.3	0.5	0.4	ND	0.3	ND	0.4	0.3
$C_{18:1}\omega9c$	0.4	0.2	0.2	0.3	0.2	0.2	ND	0.2	0.2
iso-$C_{16:0}$ 3OH	0.1	0.3	0.2	0.3	0.2	ND	ND	ND	ND
iso-$C_{13:0}$	0.5	0.2	0.2	0.4	0.3	0.9	0.3	0.2	0.2
iso-$C_{14:0}$	4.8	4.8	2.7	3.2	1.9	2.1	2.5	1.0	0.6
***iso*-$C_{15:0}$**	**31.4**	**24.4**	**23.6**	**28.6**	**44.1**	**46.6**	**33.9**	**41.3**	**33.0**
iso-$C_{15:0}$ 3OH	0.2	0.2	0.2	0.3	0.2	ND	ND	ND	ND
iso-$C_{15:1}$ F	0.8	0.9	3.5	4.9	5.9	5.3	1.1	2.4	2.4
***iso*-$C_{16:0}$**	**36.0**	**45.4**	**30.5**	**25.5**	**15.7**	**13.5**	**21.6**	**17.8**	7.9
iso-$C_{16:1}$ H	2.6	2.7	6.1	2.9	2.4	1.1	1.1	1.3	0.3
iso-$C_{17:0}$	2.7	3.7	3.0	3.0	2.9	4.2	3.7	8.2	3.2
iso-$C_{18:1}$ H	0.1	0.2	0.3	ND	ND	ND	0.5	ND	ND
anteiso-$C_{13:0}$	0.3	ND	0.2	0.2	0.2	ND	ND	0.2	0.3
anteiso-$C_{15:0}$	**11.9**	5.6	3.3	4.0	3.7	3.2	5.9	2.5	**14.9**
anteiso-$C_{17:0}$	2.2	2.2	1.7	1.6	1.2	1.7	2.5	1.8	5.3
anteiso-$C_{17:1}$ w9c	0.2	0.4	0.6	0.8	0.6	0.4	0.5	0.5	1.5
Summed Feature 3*	0.4	0.2	0.4	0.6	ND	0.4	0.9	0.7	1.3
**Summed Feature 9***	3.4	6.1	**19.4**	**16.7**	**17.4**	**13.5**	**13.9**	**16.9**	**14.7**

*Summed Features are fatty acids that cannot be resolved reliably from another fatty acid using the chromatographic conditions chosen. The MIDI system groups these fatty acids together as one feature with a single percentage of the total. Summed feature 3 comprises $C_{16:1}\omega7c$ and/or $C_{16:1}\omega6c$; summed feature 9 comprises $C_{16:0}$ 10-methyl and/or *iso*-$C_{17:1}\omega9c$.

Table S7. Differential characteristics of strains isolated from the faeces of bats

Strains: 1, *Ornithinimicrobium sufpigmenti* HY006[T]; 2, *Ornithinimicrobium faecis* HY1793[T]; 3, *Apibacter raozihei* HY039; 4, *Brevibacterium zhoupengii* HY001/HY170[T]; 5, Microbacterium fandaimingii HY82[T]; 6, *Gordonia zhenghanii* HY002[T];7, *G. liuliyuniae* HY366[T]; 8, *Tomitella gaofuii* HY086; 9, *T. fengzijianii* HY187

**Characteristics**	**1**	**2**	**3**	**4**	**5**	**6**	**7**	**8**	**9**
Colony color	yellow	white	yellow	Creamy	white	Creamy	Creamy	Creamy	Creamy
Gram-stain	Positive	Positive	Negative	Positive	Positive	Positive	Positive	Positive	Positive
Source of isolation	*Rousettus leschenaultia*	*Rousettus leschenaultia*	*Rousettus leschenaultia*	*Taphozous perforates*, *Rousettus leschenaultia*	*Rousettus leschenaultia*	*Rousettus leschenaultia*	*Rousettus leschenaultia*	*Taphozous perforates*	*Taphozous perforates*
Phylum	Actinobacteria	Actinobacteria	Bacteroidota	Actinobacteria	Actinobacteria	Actinobacteria	Actinobacteria	Actinobacteria	Actinobacteria
Temperature (°C)	15–40	15–37	14–40	4–32	10–30	15–35	15–35	10–32	15–32
Optimal temperature (°C)	28–30	28	35	25–28	28	28	28	25–28	25–28
NaCl range (w/v)	0–7.0	0–9.0	0–3.0	0–15	0.5–10.5	0–6	0–6	0–11	0–8
Optimal NaCl	1	1.0–1.5	0–0.5	0.5–1.0	0.5	0.5–1.0	0.5–1.0	2.0–3.0	2.0–3.0
pH range	7.0–11.0	6.5–10.0	5.0–9.0	5.0–10.0	6.0–10.0	5.0–10.0	5.0–10.0	5.0–10.0	5.0–9.0
Optimal pH	8.0–8.5	8	6.5	7.5	8	6.5	7	6.5–7.5	7.0–7.5
Genome size (bp)	4,169,359	4,620,106	3,296,886	4,310,443	3,622,163	4,103,234	4,102,274	4,324,277	3,991,059
G+C content (%)	71.4	68.7	32	62.7	63.3	66.5	66.9	70.9	70.9
Major fatty acids (>10%)	*iso*-$C_{15:0}$, *iso*-$C_{16:0}$ *anteiso*-$C_{15:0}$	*iso*-$C_{15:0}$, *iso*-$C_{16:0}$, Summed feature 9#	$C_{16:0}$, *iso*-$C_{15:0}$, iso- $C_{17:0}$ 3OH, Summed feature 9#	anteiso-$C_{15:0}$, anteiso-$C_{17:0}$	anteiso-$C_{15:0}$, anteiso-$C_{17:0}$, *iso*-$C_{15:0}$, *iso*-$C_{16:0}$	$C_{16:0}$, 10-methyl-$C_{18:0}$, $C_{18:1}\omega9c$, Summed feature 3#	$C_{16:0}$, 10-methyl-$C_{18:0}$, $C_{18:1}\omega9c$, Summed feature 3#	$C_{16:0}$, 10-methyl-$C_{18:0}$, $C_{18:1}\omega9c$, Summed feature 3#	$C_{16:0}$, 10-methyl-$C_{18:0}$, $C_{18:1}\omega9c$, Summed feature 3#
Major predominant menaquinone	MK-8(H_4)	MK-8(H_4)	MK-6	MK-8(H_2)	MK-11, MK-12	MK-9(H_2)	MK-9(H_2)	MK-9(H_2)	MK-9(H_2)

#Summed feature 3 comprises $C_{16:1}\omega7_C$ and/or $C_{16:1}\omega6_C$; Summed feature 9 comprises $C_{16:0}$ 10-methyl and/or *iso*-$C_{17:1}\omega9_C$.

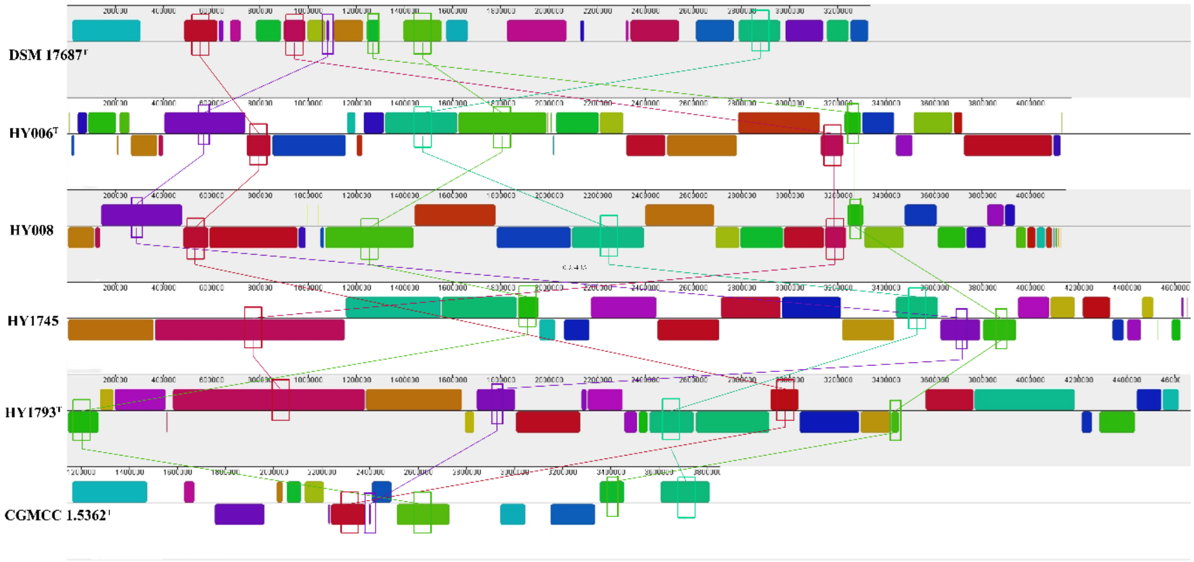

Fig. S1. Depiction of a common locally collinear blocks from the genomes of the six *Ornithinimicrobium* spp strains. Colourful rectangles and lines represent locally collinear blocks (LCBs) which are the most conserved collinear regions of the genomes.

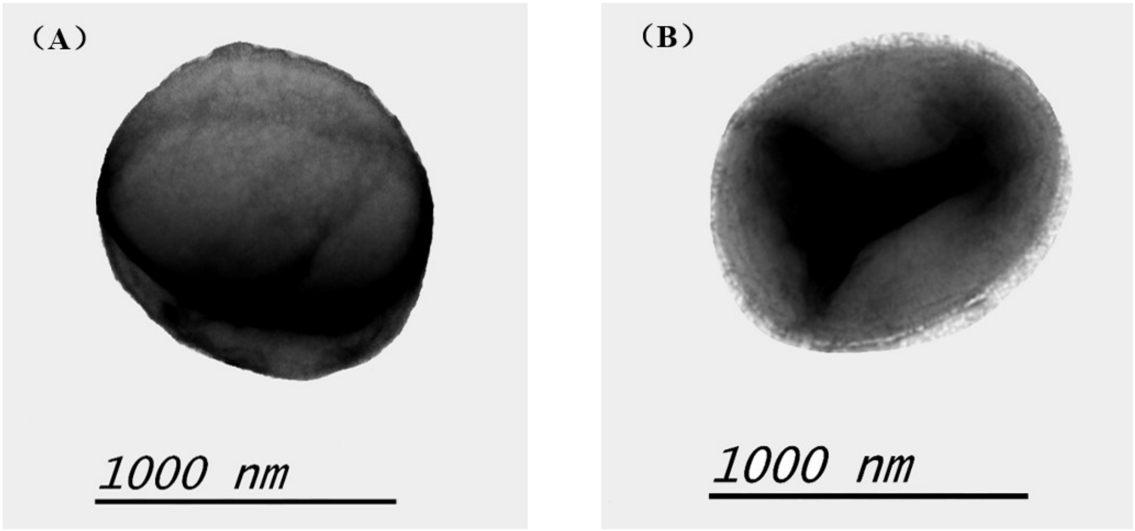

Fig. S2. Transmission electron micrograph of type strain HY006^{T} (A) and HY1793^{T} (B).

Grown on BHI-1.0% (w/v) NaCl agar at 28°C for 5 days. Bar, 1000 nm.

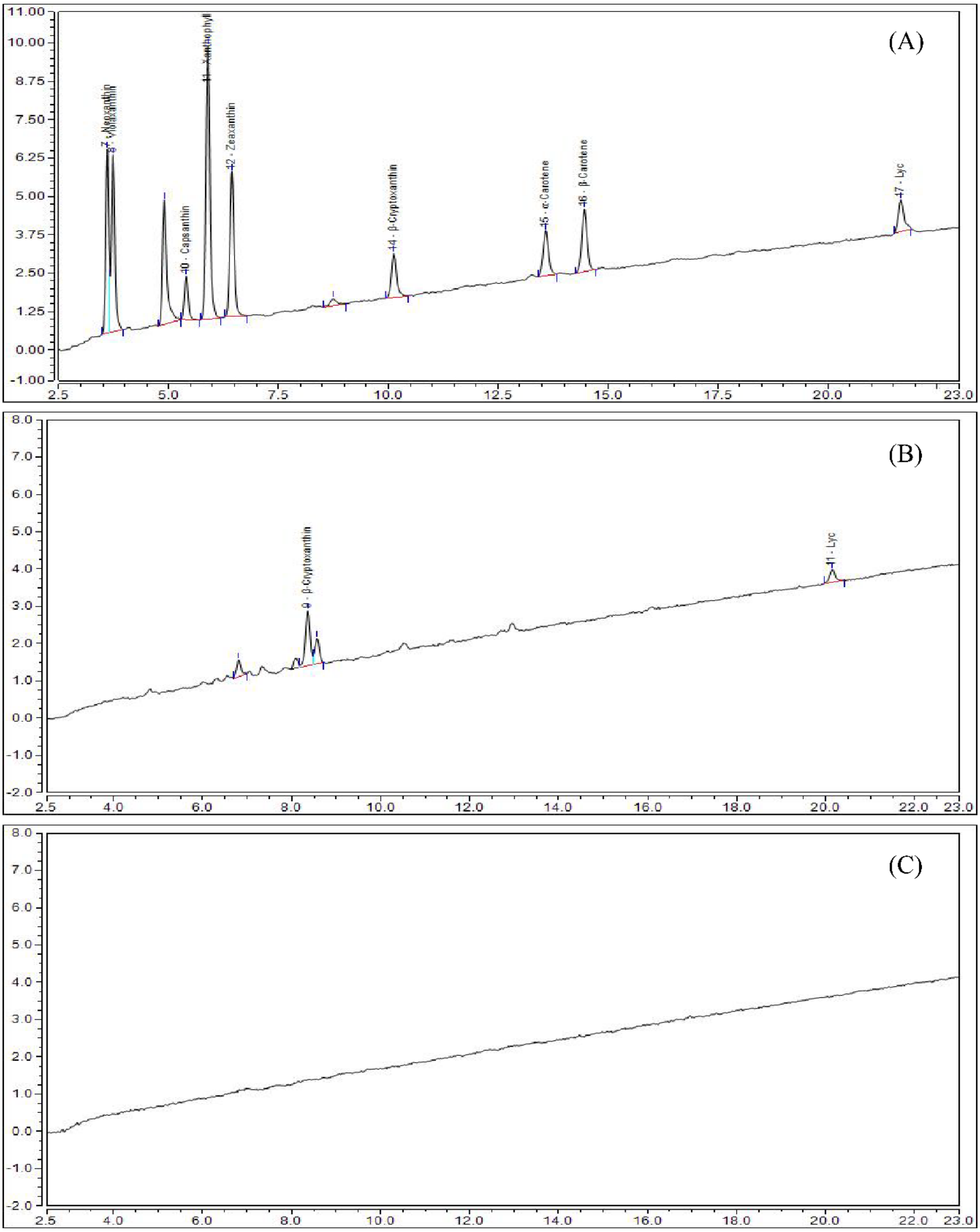

Fig. S3. The total ion chromatograms of carotenoid.

(A), calibration standard, including neoxanthin, violaxanthin, capsanthin, xanthophyll, zeaxanthin, β-cryptoxanthin, α-carotene, β-carotene and lycopene (Lyc); (B), strain HY006^{T}; (C), strain HY1793^{T}.

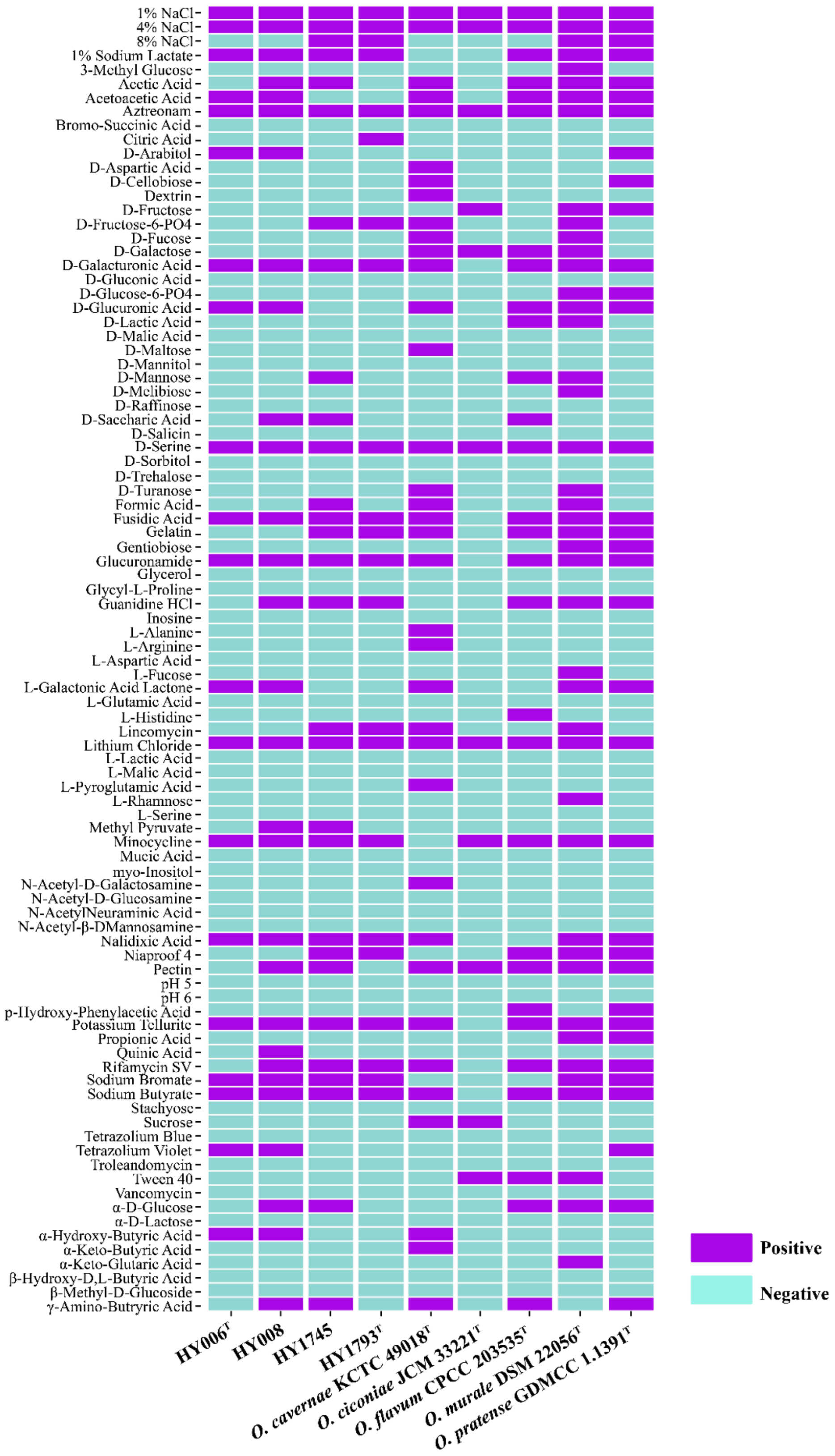

Fig. S4. Assimilation overview of the substrate sources for *Ornithinimicrobium* spp strains according to the Biolog GEN III MicroPlate.

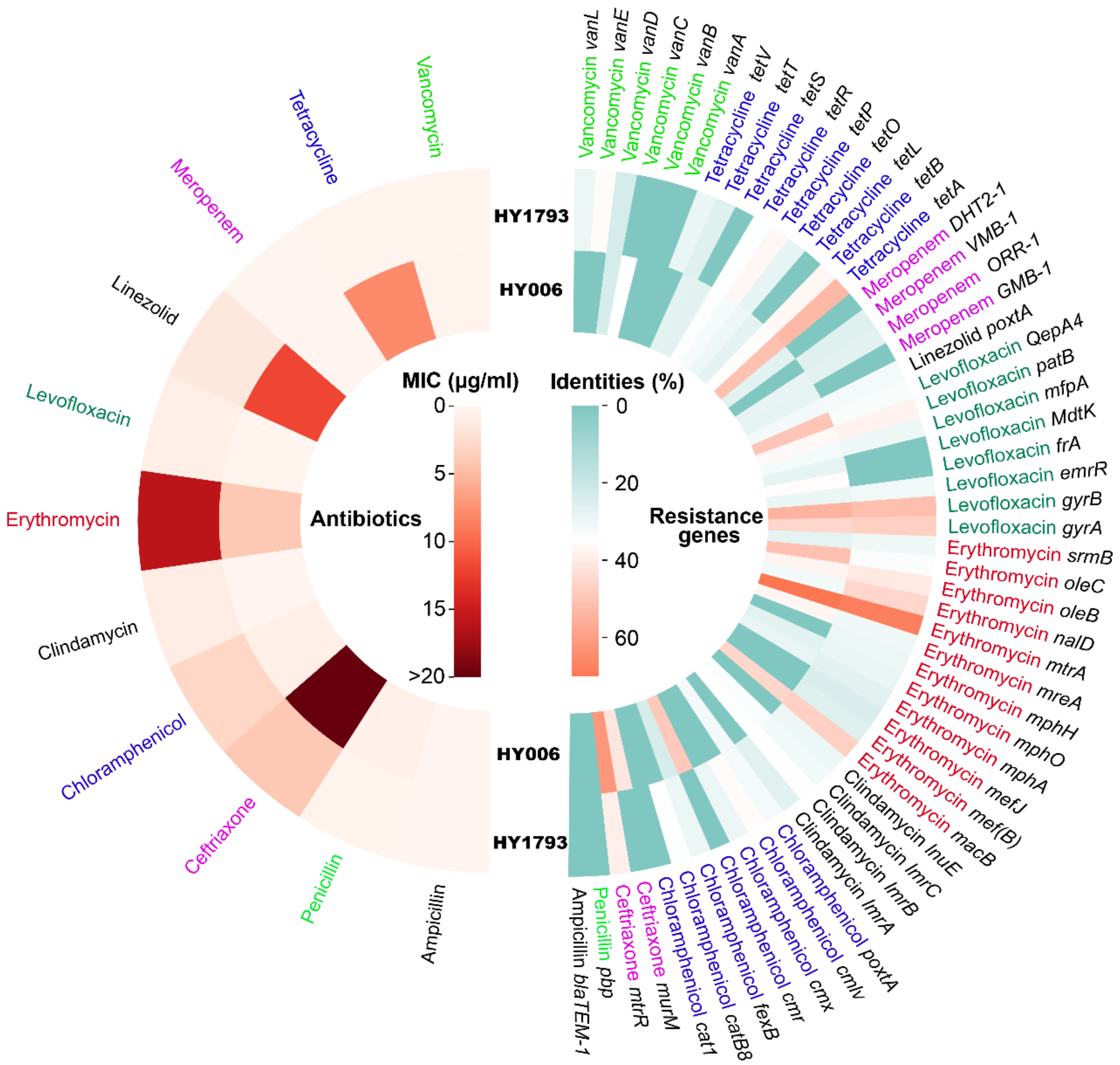

Fig. S5. Antibiotic resistance profile of strains HY006 and HY1793.

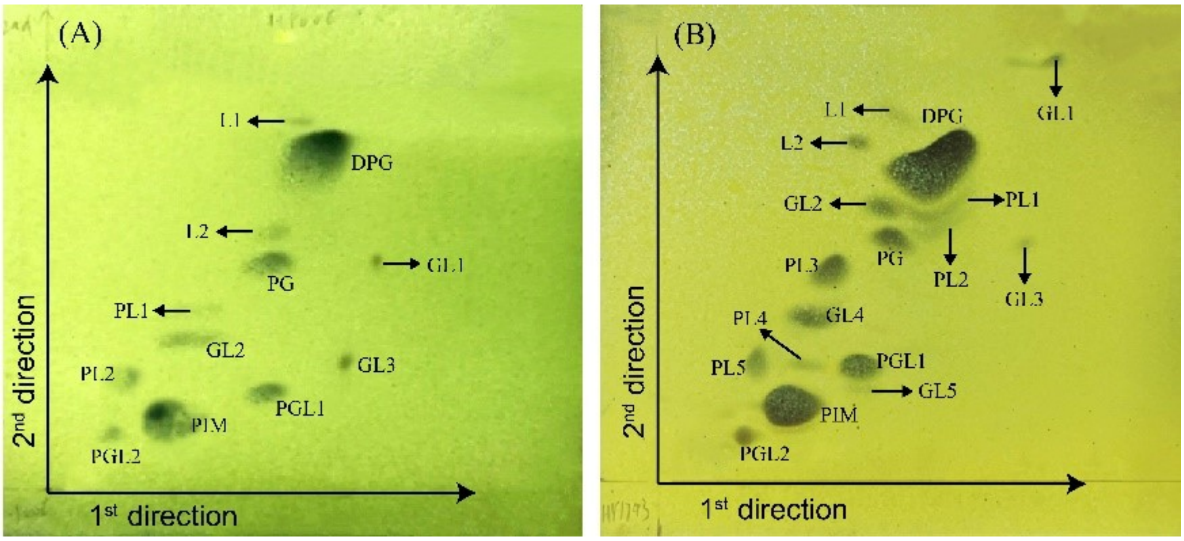

Fig. S6. Polar lipid profiles of strains HY006^T (A) and HY1793^T (B).

The solvent systems used were chloroform/methanol/water (65:25:4, v/v) for the first dimension and chloroform/methanol/acetic acid/water (80:18:12:5, v/v) for the second dimension. Abbreviations: DPG, diphosphatidylglycerol; PG, phosphatidylglycerol; PIM, phosphatidyl inositol mannoside; PGL, unknown phosphoglycolipids; PL, phospholipid; GL, unidentified glycolipids; L1-2, unidentified lipids.

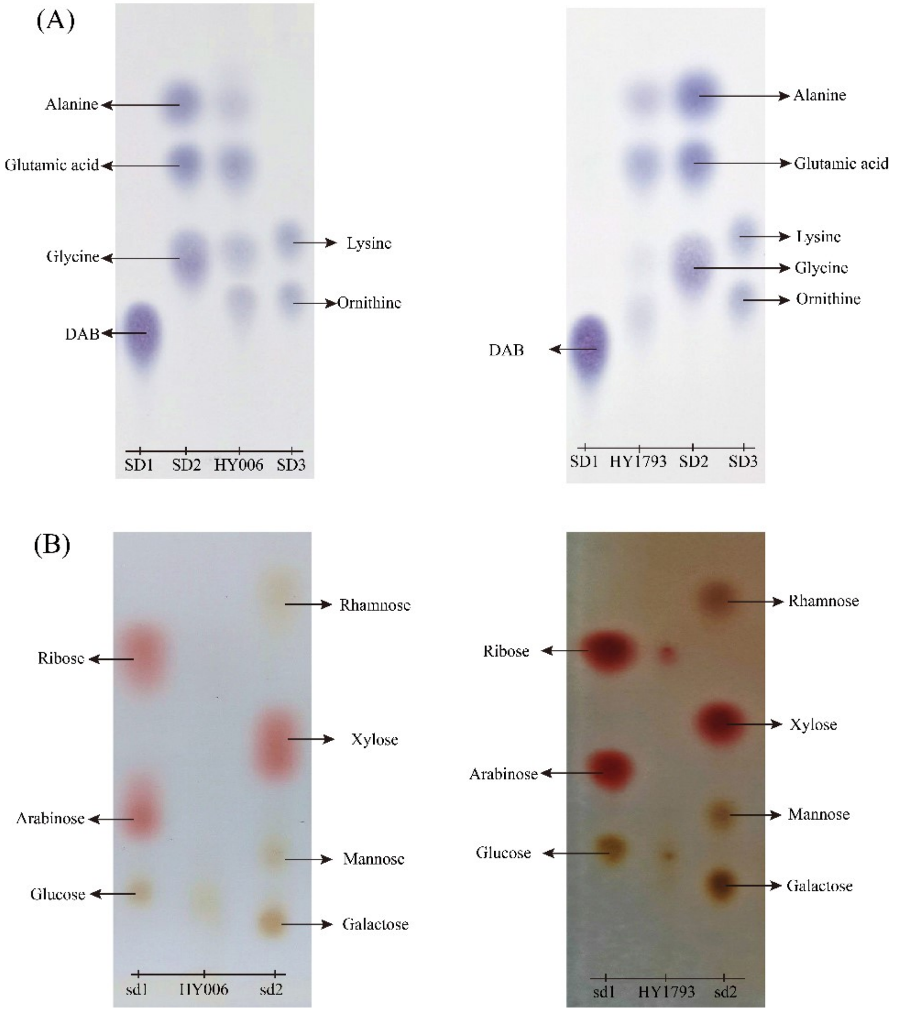

Fig. S7. The amino acid (A) and sugar (B) composition of strains HY006^T and HY1793^T.

Abbreviations: SD, amino acid standards; sd, sugar standards.
